# Exploring Human Brain Metabolism via Genome-Scale Metabolic Modeling with Highlights on Multiple Sclerosis

**DOI:** 10.1021/acschemneuro.5c00006

**Published:** 2025-03-17

**Authors:** Mustafa Sertbas, Kutlu O. Ulgen

**Affiliations:** † Department of Chemical Engineering, Bogazici University, 34342 Istanbul, Turkey; ‡ Department of Chemical Engineering, Istanbul Technical University, 34469 Istanbul, Turkey

**Keywords:** brain metabolism, genome-scale metabolic modeling, flux balance analysis, reporter metabolites, multiple sclerosis

## Abstract

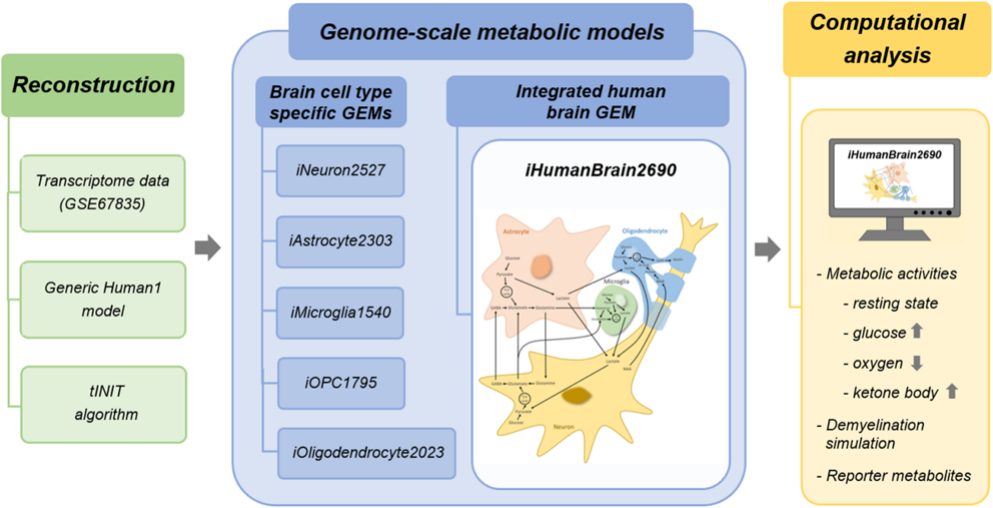

Cerebral dysfunctions give rise to a wide range of neurological diseases due to the structural and functional complexity of the human brain stemming from the interactive cellular metabolism of its specific cells, including neurons and glial cells. In parallel with advances in isolation and measurement technologies, genome-scale metabolic models (GEMs) have become a powerful tool in the studies of systems biology to provide critical insights into the understanding of sophisticated eukaryotic systems. In this study, brain cell-specific GEMs were reconstructed for neurons, astrocytes, microglia, oligodendrocytes, and oligodendrocyte precursor cells by integrating single-cell RNA-seq data and global Human1 via a task-driven integrative network inference for tissues (tINIT) algorithm. Then, intercellular reactions among neurons, astrocytes, microglia, and oligodendrocytes were added to generate a combined brain model, iHumanBrain2690. This brain network was used in the prediction of metabolic alterations in glucose, ketone bodies, oxygen change, and reporter metabolites. Glucose supplementation increased the subsystems’ activities in glycolysis, and ketone bodies elevated those in the TCA cycle and oxidative phosphorylation. Reporter metabolite analysis identified L-carnitine and arachidonate as the top reporter metabolites in gray and white matter microglia in multiple sclerosis (MS), respectively. Carbamoyl-phosphate was found to be the top reporter metabolite in primary progressive MS. Taken together, single and integrated iHumanBrain2690 metabolic networks help us elucidate complex metabolism in brain physiology and homeostasis in health and disease.

## Introduction

Cerebral dysfunctions affect millions of individuals across all regions of the world and give rise to a wide range of neurological diseases due to the structural and functional complexity of the human brain stemming from the interactive cellular metabolism of its specific cells including neurons and glial cells (astrocytes, microglia, and oligodendrocytes).^1^ Neurons transmit electrical and chemical signals to different parts of the body. Astrocytes provide regulation of the brain’s environment and metabolic support to neurons. Microglia are resident macrophages in the central nervous system (CNS). Oligodendrocytes produce a myelin sheath that wraps adjacent axons of neurons. These brain-specific cells perform altered adaptive responses to metabolic stresses and bioenergetic challenges in health, aging, and neurodegeneration.^2−4^

Advances in isolation and measurement technologies have provided a comprehensive understanding of cerebral events in health and disease at a single-cell resolution.^5,6^ The transcriptional differences between brain-specific cells were also elucidated at the single-cell level.^7,8^ For example, the Alzheimer’s disease (AD) risk gene *APOE* was downregulated in oligodendrocytes and astrocytes; however, it was upregulated in the microglia of patients with AD.^8^ Moreover, the transcriptional profiling of human microglia elucidated heterogeneity between gray matter (GM) and white matter (WM) in multiple sclerosis (MS), which is the most prevalent nontraumatic debilitating illness affecting young individuals.^9,10^ The pathological hallmark of MS includes inflammatory lesions, demyelination, and damaged oligodendrocytes. It occurs when the immune system mistakenly attacks the protective myelin sheath covering nerve fibers, causing inflammation and damage. This disrupts the communication between the brain and the rest of the body, leading to various symptoms. The exact cause is unknown, but genetic predisposition, environmental factors, and infections may contribute. The treatment strategies such as disease-modifying therapies, physical therapy, and symptomatic management help MS patients to slow progression and improve quality of life.^11^ In addition to oligodendrocytes, astrocytes are also recognized as highly active players during lesion formation in MS.^12^ Understanding the roles of these various brain cell types is crucial for developing targeted therapies aimed at modulating the immune response, protecting myelin, and promoting repair in neurodegenerative diseases such as MS.

Computational approaches provide critical insight into the understanding of prokaryotic and eukaryotic systems. In this regard, genome-scale metabolic models (GEMs), which cover the metabolic reaction network of cellular metabolism with related genes and enzyme information, have become a powerful tool in the studies of systems biology over the past two decades. In addition to global human GEMs, tissue-specific GEMs were reconstructed to investigate certain tissue metabolism in health and disease.^13−17^ Especially when considering the inherent difficulty of experimental studies in the human brain, metabolic interactions and neurotransmitter metabolism in and between astrocytes and neurons were investigated by brain-specific GEMs at the system level.^18−21^

In context-specific modeling, generic human GEMs are adapted to specific conditions, tissues, or diseases by incorporating experimental omics data such as transcriptomics, proteomics, or metabolomics.^22,23^ This method captures unique metabolic activity within a given biological context. By providing an overview of metabolism at the system level, context-specific GEMs help uncover the molecular basis of complex phenotypes and improve personalized medicine strategies. Utilizing a context-specific GEM can be a valuable approach for studying MS from a systems biology perspective, providing insights into its complex pathology and exploring potential avenues for therapeutic intervention. The metabolic pathways dysregulated in MS, such as changes in energy metabolism, lipid metabolism, and neurotransmitter synthesis, can be captured by context-specific GEMs.

Current brain-specific GEMs include astrocyte and neuron compartments. However, microglia and oligodendrocytes also play a critical role in brain physiology and homeostasis in health and disease.^3,24−29^ Here, brain cell-specific GEMs were reconstructed for neurons, astrocytes, microglia, oligodendrocytes, and oligodendrocyte precursor cells (OPCs) by integrating single-cell RNA-seq data and global Human1.^7,30^ The integration of expression data was carried out by a task-driven integrative network inference for tissues (tINIT) algorithm.^31^ Then, transport reactions between these specific cells were added to represent their crosstalk in the brain. Then, iHumanBrain2690 was employed to estimate the metabolic alterations in response to changes in glucose, ketone bodies, and oxygen supplies, occurring in several neurodegenerative diseases, and to predict reporter metabolites in MS. The reconstruction of single-cell-level GEMs for astrocytes, neurons, microglia, and oligodendrocytes and their integration into a combined brain model iHumanBrain2690 decipher cell-specific key features of different metabolic states and diseases such as neurodegenerative and neurodevelopmental disorders as well as intracranial tumors.

## Results and Discussion

### Reconstructed Cell Type-Specific and Integrated Human Brain GEMs

**Figure 1:**
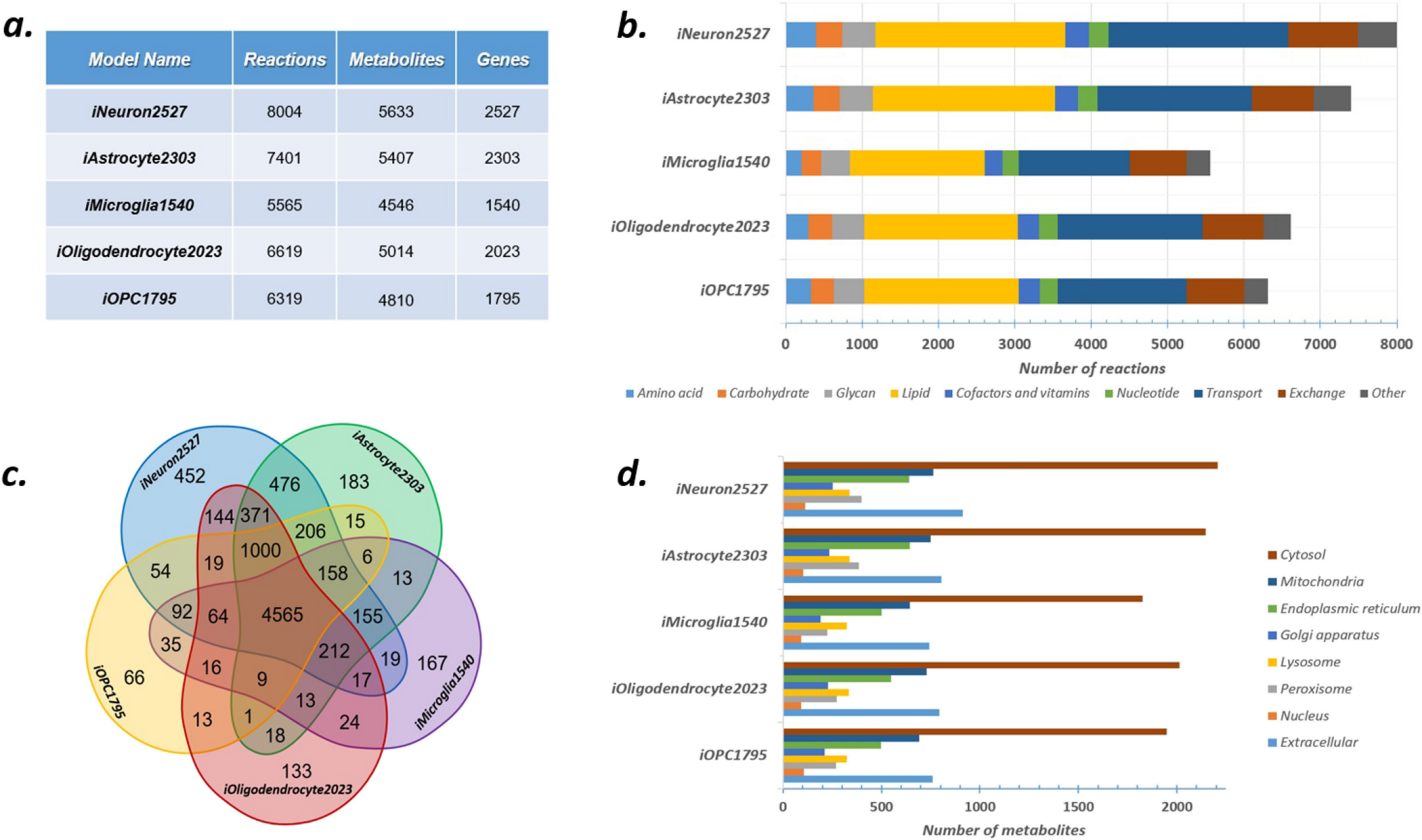
(a) Comparison of the number of reactions, metabolites, and genes, (b) number of reactions in different metabolisms, (c) number of shared reactions, and (d) distribution of metabolites in the cellular compartments in reconstructed cell type-specific human brain GEMs.

**Figure 2:**
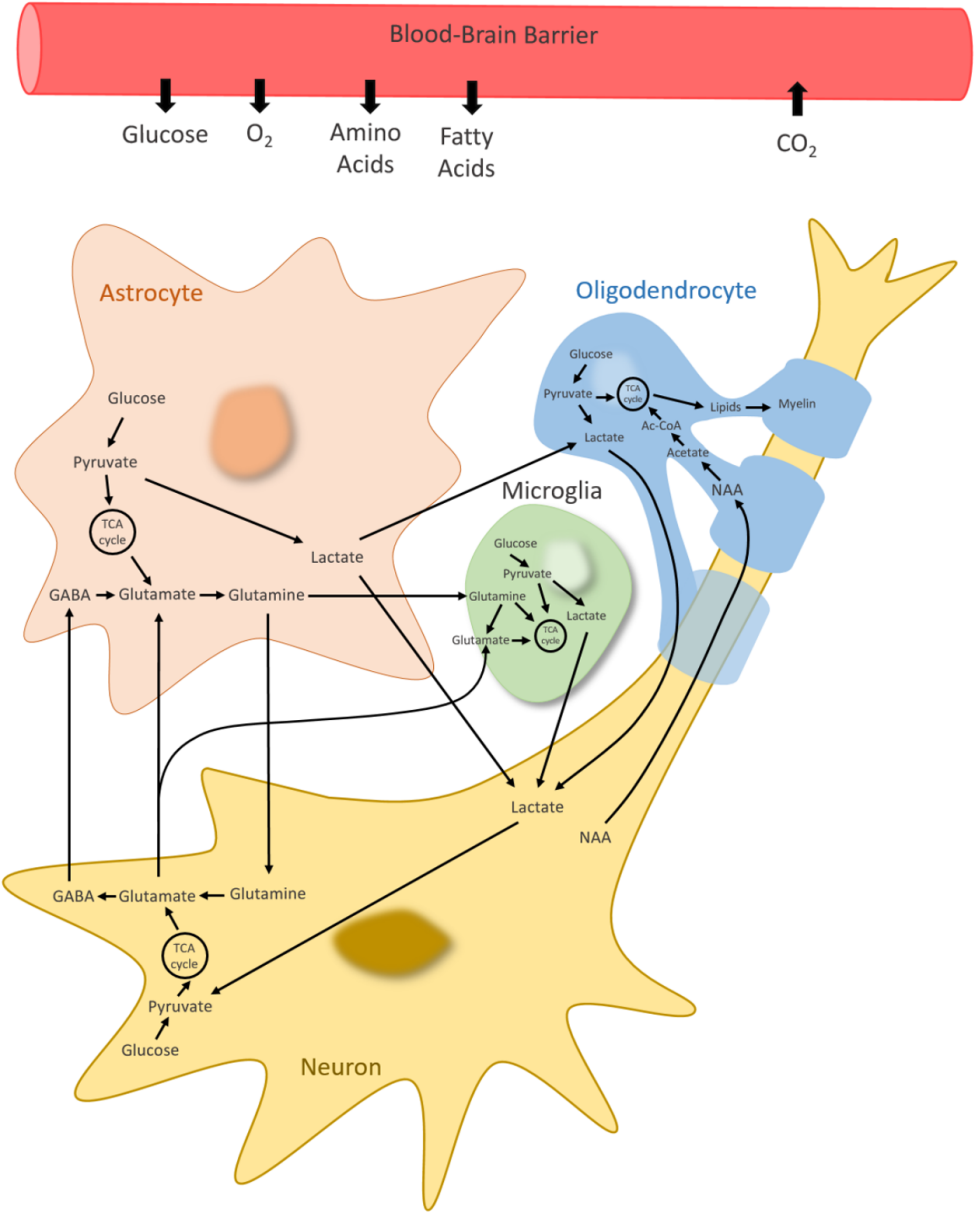
Metabolic crosstalk in iHumanBrain2690.

For the investigation of the brain-specific cellular metabolism, five GEMs (iNeuron2527, iAstrocyte2303, iMicroglia1540, iOligodendrocyte2023, and iOPC1795) were reconstructed for brain cell types including neuron, astrocyte, microglia, oligodendrocyte, and OPC (Supplementary Data 1) based on their single-cell RNA sequencing and tINIT algorithm. Along with 57 essential metabolic tasks, brain cell type-specific reactions were used as essential tasks in the reconstruction of GEMs. The highest and least number of reactions, metabolites, and genes were identified in the iNeuron2527 and iMicroglia1540 metabolic networks (Figure 1a), respectively. The distribution of reactions resulted in lipid metabolism, with the highest number of reactions in all reconstructed GEMs (Figure 1b). 4565 metabolic reactions were found to be in common in all generated metabolic models (Figure 1c). Metabolite distribution demonstrated that cytosol is the top subcellular compartment with the highest number of metabolites in all human brain cell-specific GEMs (Figure 1d). Metabolic task performances of all GEMs were evaluated by 257 metabolic tasks listed by Robinson et al. (2020).^30^ iNeuron2527 and iMicroglia1540 successfully performed 218 and 169 metabolic tasks, respectively (Table S1). Subsequent to the generation of single brain cell-specific GEMs, integration of neurons, astrocytes, microglia, and oligodendrocyte compartments into whole human brain GEM was performed by intercellular reactions which represent metabolic trafficking among them. The final integrated human brain GEM, called as iHumanBrain2690, includes 27615 metabolic reactions, 20600 metabolites, and 2690 genes (Figure 2).

### Metabolic Activities in Single Brain Cells and Whole Brain iHumanBrain2690 at Resting State

**Figure 3:**
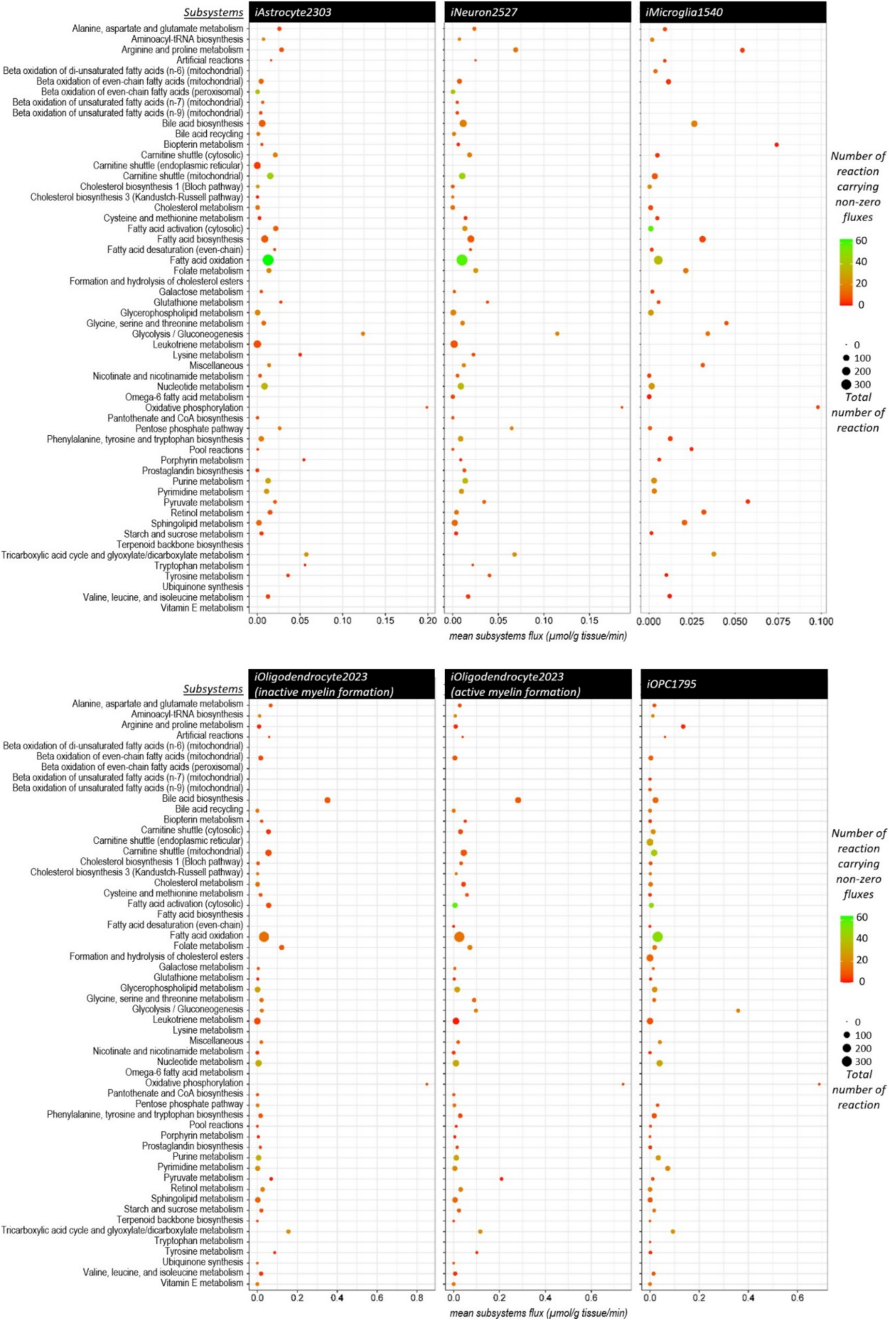
Mean subsystem fluxes in reconstructed brain cell type-specific GEMs at resting state.

The flux of each metabolic reaction was minimized and maximized as an objective function for flux variability analysis (FVA) (Supplementary Data 1). The flux balance analysis (FBA) was performed for single brain cells with the maximization of biomass reaction flux as the objective function to computationally elucidate metabolic activities in a healthy-state human brain (Figure 3) (Supplementary Data 1). The maximization of biomass reaction predicted the fluxes of growth varying between 0.0064 μmol/g tissue/min for microglia and 0.0433 μmol/g tissue/min for OPC (Table S2). Oligodendrocyte simulation with maximization of both biomass and myelin reaction resulted in a flux value of 0.0422 μmol/g tissue/min, which is also the value for the maximization of only its biomass reaction flux with zero myelin reaction flux. The maximization of only myelin reaction brought about a flux value of 0.0193 μmol/g tissue/min. By setting myelin reaction flux between 0 and 0.0193 μmol/g tissue/min via upper and lower limits, the change of oligodendrocyte biomass reaction flux with myelin reaction flux was investigated, and increased myelin production resulted in decreased biomass flux as expected (Figure S1). Two different FBA simulations were performed with iOligodendrocyte2023 at myelin reaction fluxes of 0 and 0.0100 μmol/g tissue/min. Zero flux indicates inactive myelin reaction and maximum biomass flux. To keep both myelin and biomass reactions active in oligodendrocytes, myelin formation flux was set to 0.0100 μmol/g tissue/min in which biomass reaction flux is 0.0278 μmol/g tissue/min.

The number of nonzero fluxes (flux value >10^–6^ μmol/g tissue/min) changes between 944 (microglia) and 1664 (neuron) in healthy-state simulation (Table S2). Mean subsystem fluxes were calculated based on the reactions with nonzero fluxes, and the subsystems with a minimum of 5 nonzero fluxes in any generated GEM resulted in 56 subsystems (Figure 3) excluding transport and exchange/demand reactions (Table S3). Despite consisting of a small number of metabolic reactions, oxidative phosphorylation has the highest mean fluxes in all brain cell types. Fatty acid oxidation, which includes the greatest number of total reactions, has more than 50 reactions with nonzero fluxes in neurons, astrocytes, and OPCs.

Glucose is the primary substrate in the energy metabolism of astrocytes, neurons, microglia, and oligodendrocytes.^28,32,33^ It is taken up from the blood circulation and metabolized to pyruvate via glycolysis. Pyruvate can either be converted to lactate in the cytosol by anaerobic respiration or enter the mitochondria to be further metabolized via the TCA cycle and oxidative phosphorylation. Long-chain fatty acids are transported into mitochondria via a carnitine shuttle for β-oxidation and energy production. Fatty acyl CoA is β-oxidized into acetyl-CoA which is metabolized through the TCA cycle and oxidative phosphorylation to generate ATP. Fatty acid and cholesterol biosynthesis start with acetyl-CoA to form malonyl-CoA and acetoacetyl-CoA, respectively.^34,35^ In fatty acid metabolism, acetyl-CoA and malonyl-CoA are converted to palmitic acid. Then, elongation and desaturation reactions form polysaturated and unsaturated fatty acids. In cholesterol metabolism, acetoacetyl-CoA and acetyl-CoA are catalyzed to 3-hydroxy-3-methylglutaryl-CoA (HMG-CoA). It is converted into mevalonate, a key intermediate in cholesterol biosynthesis.

**Figure 4:**
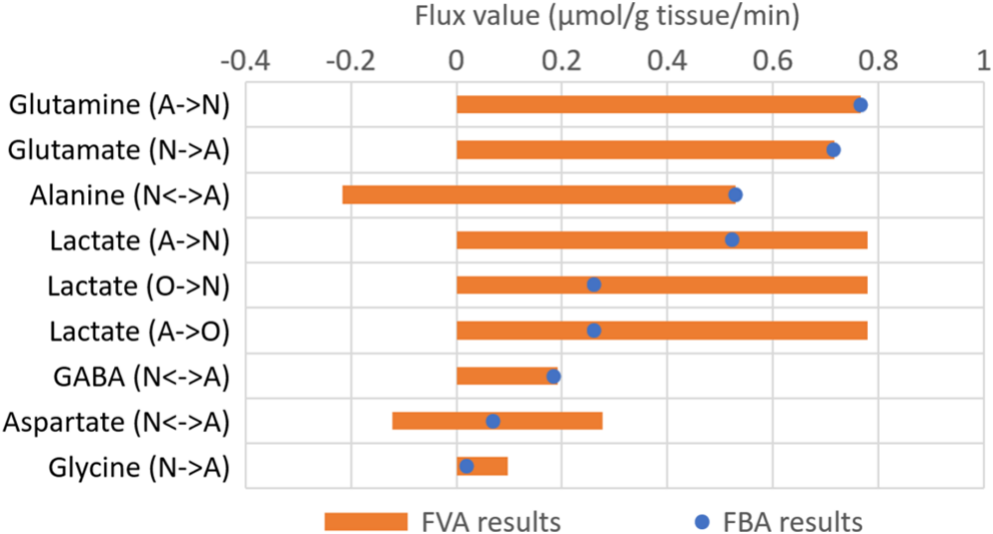
Intercellular reaction fluxes in iHumanBrain2690 at the resting state.

Subsequent to single-cell predictions, FBA and FVA were applied with the maximization of the glutamate/glutamine/GABA cycle between neuron and astrocyte in the integrated iHumanBrain2690. The exchange reaction fluxes obtained from single-cell simulations were applied as constraints. The biomass reaction fluxes and number of reactions with nonzero flux demonstrated similar results with the single-cell computations (Table S4). Also, the computation of intercellular transfer reactions, including glutamate/glutamine/GABA cycle, glycine, aspartate, alanine, and lactate reactions, resulted in fluxes greater than 0.01 μmol/g tissue/min (Figure 4). Brain-specific cells perform unique metabolite and neurotransmitter trafficking for information processing. Glutamate is the primary neurotransmitter and hub metabolite linking glucose and amino acid metabolism in brain-specific cells.^36^ Glutamate homeostasis and recycling between neurons and astrocytes via the glutamate–glutamine cycle are critical for energy metabolism and normal functioning of the brain. Glutamine is widely distributed in CNS and is a precursor of glutamate, aspartate, and GABA neurotransmitters.^37^ It is produced from glutamate and ammonia in astrocytes and transported into neurons, where it is converted into glutamate. GABA is also synthesized in neurons. Both glutamate and GABA are transferred from neurons to astrocytes. Computational analysis revealed active intercellular reaction fluxes through the glutamate/glutamine/GABA cycle in iHumanBrain2690 (Figure 4). Similarly, intercellular lactate transports among neurons, astrocytes, and oligodendrocytes were also computed as active in the iHumanBrain2690 network. Astrocyte- or oligodendrocyte-derived lactate can be taken up by neurons where it is used as an energy substrate.^28^ Lactate acts as a metabolic substrate for active neurons and has been shown to increase synaptic plasticity, decrease excitability in neurons and neural networks, have neuroprotective effects, and lessen neuroinflammation.^38^

### Metabolic Alterations in Ketogenic Diet at Resting State

**Figure 5:**
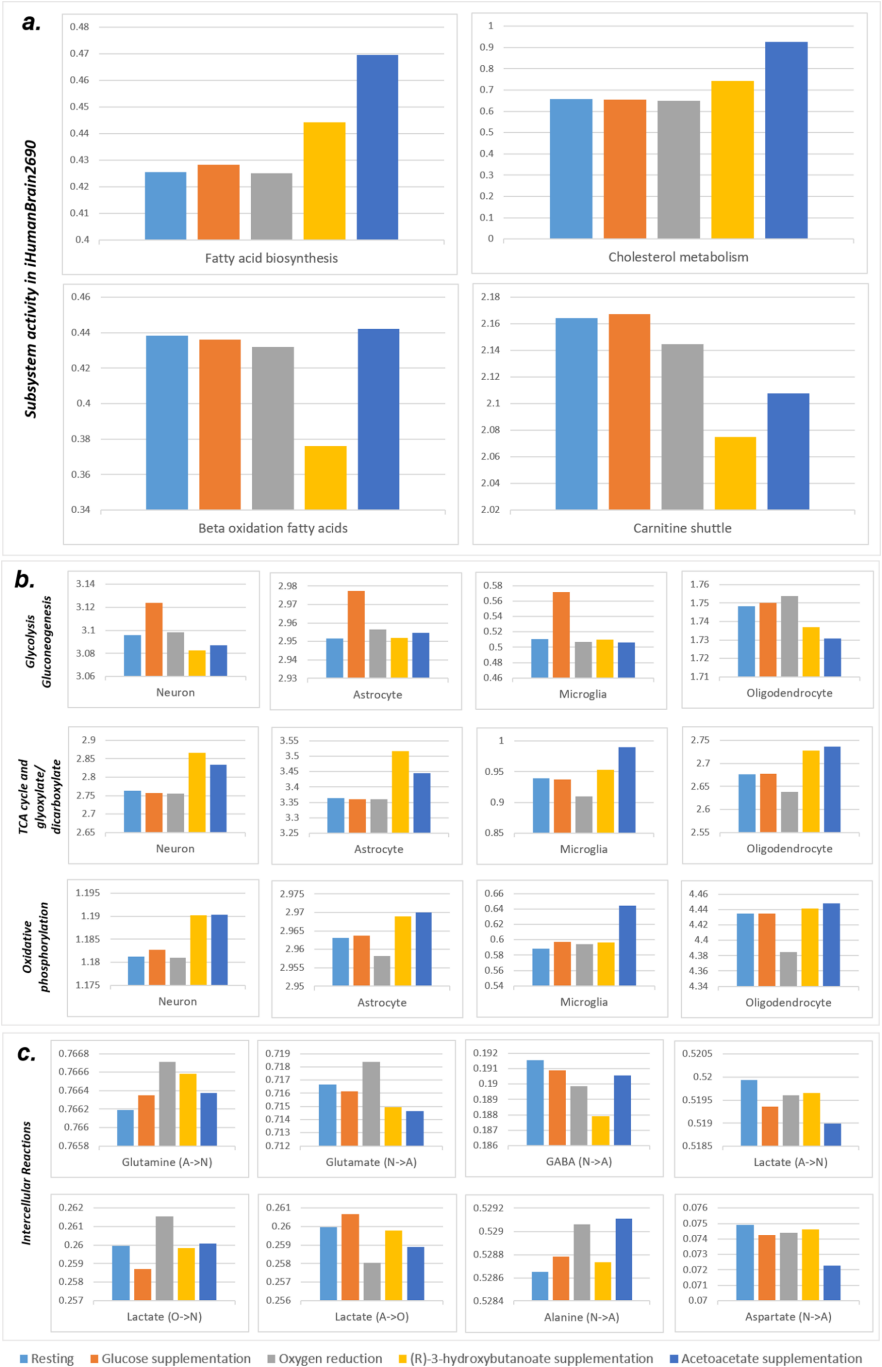
Metabolic activity alterations due to glucose and ketone body supplementations and oxygen reduction in (a) iHumanBrain2690 subsystems, (b) single brain cell subsystems, and (c) intercellular reactions. All *y*-axes demonstrate the sum of metabolic fluxes (μmol/g tissue/min) in the related subsystems.

Rather than depending on sugar (glucose) derived from carbohydrates, the keto diet utilizes ketone bodies, which are a fuel source produced by the liver from stored fat. Acetoacetate and (R)-3-hydroxybutanoate are two main ketone bodies that can substitute for glucose as alternative energy sources in the brain metabolism.^39^ ATP generation in brain cells depends on glycolysis in the cytosol and mitochondrial TCA cycle and oxidative phosphorylation. Glucose is broken down to pyruvate in glycolysis. Then, pyruvate is converted into acetyl-CoA and enters the TCA cycle. Contrary to glucose, ketone bodies are directly converted into acetyl-CoA and enter the TCA cycle without glycolysis. These metabolic processes were investigated in iHumanBrain2690 by enhancing glucose and ketone body uptake *in silico*. An increase in the acetoacetate and (R)-3-hydroxybutanoate uptake fluxes resulted in a greater TCA cycle and lower glycolysis subsystem fluxes than that of the glucose increase in four single cells in the integrated brain network (Figure 5). Thus, our reconstructed iHumanBrain2690 metabolic model successfully predicted main energy alterations in the case of glucose and ketone body supplementation. Furthermore, the response of iHumanBrain2690 to oxygen reduction pointed out a decreased trend in the TCA cycle and glyoxylate/dicarboxylate and oxidative phosphorylation subsystems activity and an increase in glycolysis/gluconeogenesis. These alterations computationally demonstrate a metabolic shift from mitochondrial aerobic metabolism to anaerobic glycolysis in the integrated human brain model due to hypoxia. Oxygen level plays an important role in cellular metabolism and its reduction diminishes oxidative phosphorylation and Krebs cycle rates.^40^ It is also critical for the generation of nitric oxide and reactive oxygen species, leading to respiration rate decrease and cellular damage, respectively. The cells adapt to prolonged hypoxia by activating hypoxia-inducible factors.^40^

In addition to the TCA cycle, acetyl-CoA relates to critical metabolic pathways including fatty acid biosynthesis, cholesterol biosynthesis, and β-oxidation of fatty acids.^41^ Metabolic activities in cholesterol metabolism and fatty acid biosynthesis were increased with the uptake of ketone bodies into the iHumanBrain2690 metabolic network (Figure 5). Owing to the supply of acetyl-CoA derived from acetoacetate and (R)-3-hydroxybutanoate, the sum of the absolute values of fluxes in β-oxidation fatty acids subsystems declined in line with the reduction in the carnitine shuttle subsystem which transports long-chain fatty acids for β-oxidation. Ketone bodies serve as significant brain energy sources and are precursors for the production of amino acids and lipids, particularly cholesterol.^42^ Brain ketone body metabolism is regulated by its blood concentration and blood–brain barrier permeability. High concentrations are observed during fasting and high-fat diets. Ketone bodies enter the mitochondrial metabolism and are used in the ATP generation and oxidative processes.^39^ To enhance brain energy metabolism with ketones, ketogenic therapies have the potential to treat various neurological diseases, including epilepsy, Alzheimer’s disease, and Parkinson’s disease.^43,44^

### Neuron and Astrocyte Support to Oligodendrocytes in Demyelination

As our simulations are in agreement with the experimental results published in the literature, validating the correct reconstruction of our integrated metabolic brain GEM, iHumanBrain2690, we next applied this model to myelin degradation to understand the key systemic effects on brain metabolism. Damage to the protective myelin sheath around nerve cells is a common feature of demyelinating diseases. To investigate demyelination metabolism via genome-scale metabolic modeling, the myelin formation flux in the oligodendrocyte compartment of the iHumanBrain2690 was reduced from 0.010 μmol/g tissue/min to zero at 10% intervals. The metabolic flux alterations were investigated in four brain cells using the minimization of metabolic adjustment (MOMA) approach interrelatedly. Not only oligodendrocyte metabolism but also neuron, astrocyte, and microglia metabolisms were altered in the iHumanBrain2690 network.

**Figure 6:**
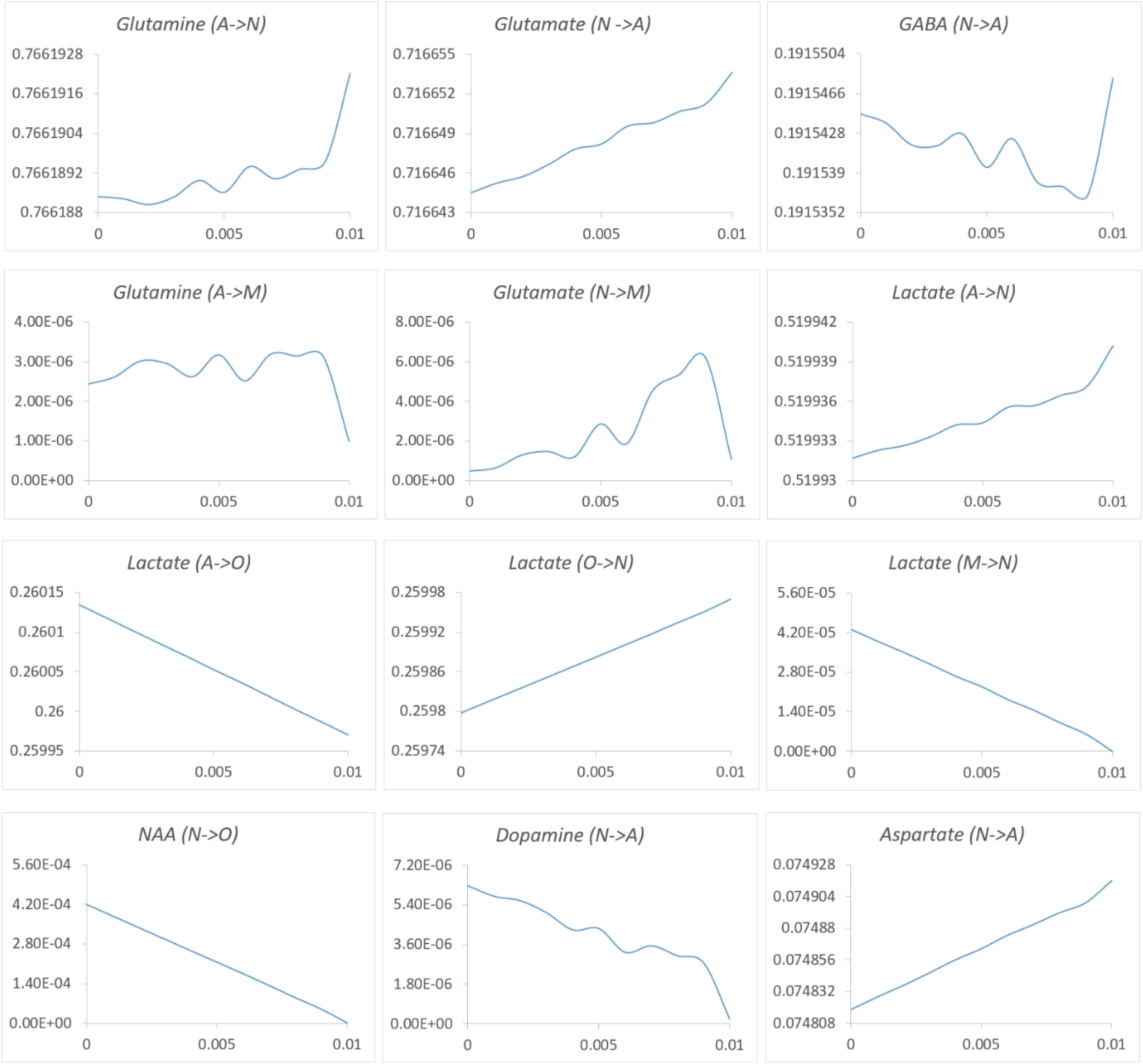
Change of metabolic fluxes (μmol/g tissue/min) among neurons, astrocytes, microglia, and oligodendrocytes in the iHumanBrain2690 network due to demyelination. All *x*-axes represent the myelin reaction flux (μmol/g tissue/min) in oligodendrocytes.

Neurotransmitters play a crucial role in brain homeostasis in health and disease. The flux alterations of intercellular reactions computationally demonstrated neurotransmitters and metabolites trafficking between single brain cells due to demyelination (Figure 6). Glutamate and GABA are primary excitatory and inhibitory neurotransmitters in CNS, respectively. In demyelination, glutamate and GABA transfers from neuron to astrocyte were decreased, and glutamate transport into microglia was increased in the iHumanBrain2690 network (Figure 6). Experimental studies reported altered levels of these neurotransmitters and suggested glutamate and GABA metabolisms as therapeutic targets in the most common demyelinating disease MS.^45^ Moreover, in MS, when astrocytes are stimulated by cytokines, their homeostatic and metabolic functions are reduced, leading to impaired glutamate uptake. This impairment can result in excitotoxicity and cause a metabolic disconnect from axons/neurons due to a decreased release of lactate.^12^ Glutamine transport from astrocytes into neurons was also reduced (Figure 6). Hence, the total flux of glutamate/glutamine/GABA cycle between astrocyte and neuron was declined due to demyelination. The flux of neurotransmitter aspartate into astrocytes was decreased in computational analysis. Dietary supplements of d-aspartate enhanced synaptic plasticity reserve and decreased fatigue in patients with progressive MS.^46^

Neurons and astrocytes provide N-acetyl-l-aspartate (NAA) and lactate to oligodendrocytes (Figure 6). NAA is a notable metabolite that transfers from neurons to oligodendrocytes. NAA, which is synthesized from acetyl-CoA and aspartate, is converted to acetate and aspartate. They are utilized in fatty acid synthesis, protein synthesis, and different metabolic processes in the cells. NAA production flux in neurons and transport into oligodendrocytes were elevated in myelin degradation using the iHumanBrain2690 metabolic network. The conversion of NAA into aspartate and acetate in oligodendrocytes was also increased, and NAA-derived aspartate and acetate participated in cellular processes due to demyelination. Experimental studies demonstrated reductions in NAA levels in GM and WM due to axonal injury in MS.^47^ Lactate transport among brain cells was also altered due to demyelination in oligodendrocytes. Lactate transport from astrocyte to oligodendrocyte was enhanced, and its transport from oligodendrocyte to neuron was declined. These demonstrated a requirement for lactate in oligodendrocytes for cellular activities in demyelination. Serum lactate levels in patients with MS were measured higher than that of healthy controls and it was suggested as a potential biomarker in MS.^48^

**Figure 7:**
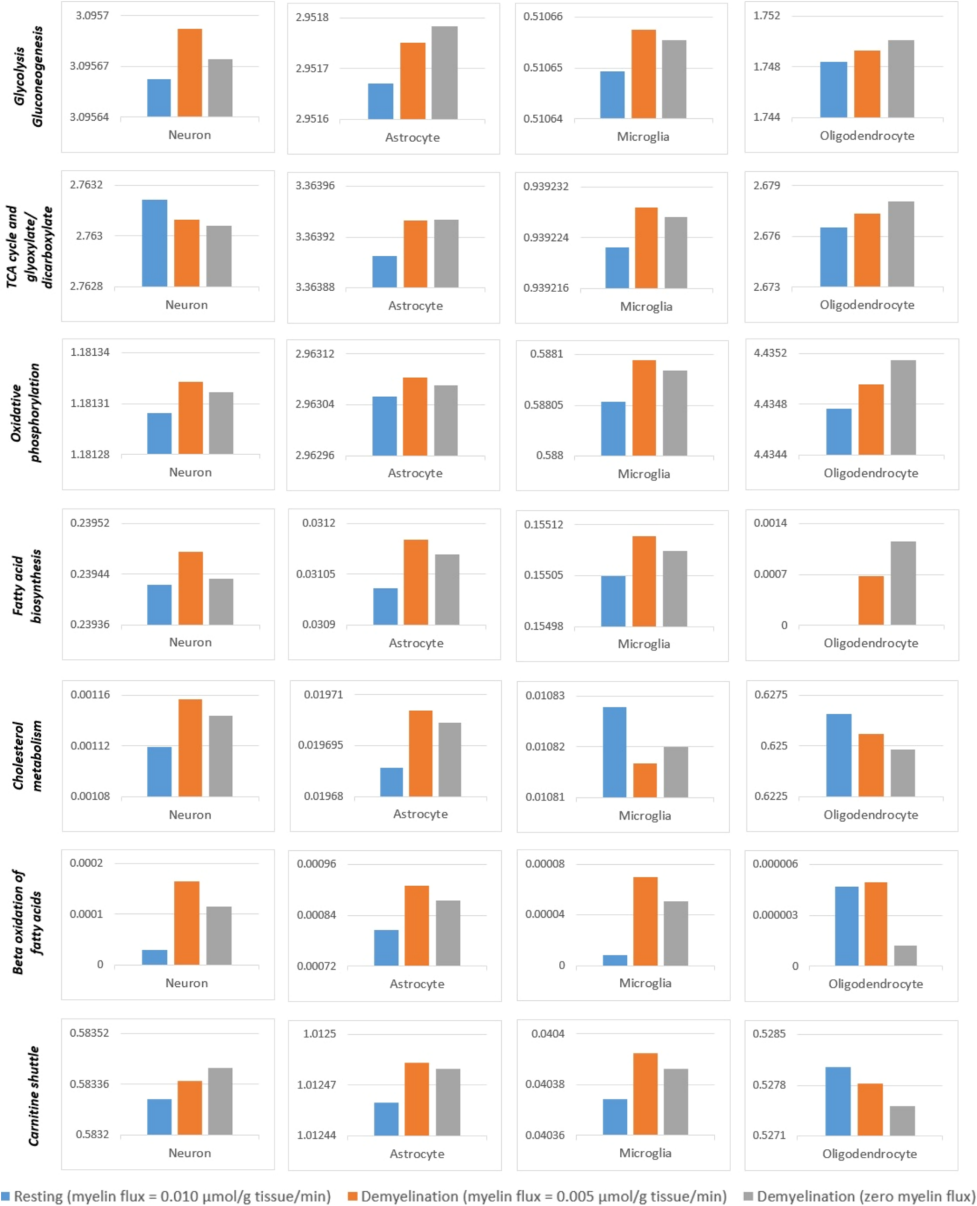
Change of metabolic activities in brain cell types in iHumanBrain2690 due to demyelination. All *y*-axes demonstrate the sum of metabolic fluxes (μmol/g tissue/min) in the related subsystems.

Metabolic activities in demyelination were computationally investigated by decreasing myelin formation flux in oligodendrocytes, and the sum of metabolic fluxes resulted in various subsystem responses in different brain cell types in iHumanBrain2690 (Figure 7). The total metabolic fluxes of glycolysis and oxidative phosphorylation subsystems were enhanced in all brain cell types. However, in contrast to responses of astrocytes, microglia, and oligodendrocytes, TCA cycle metabolic activity was only decreased in the neuron compartment of iHumanBrain2690. The brain of MS patients demonstrates impaired glucose metabolism.^49^ An experimental study on MS animal model astrocytes revealed a significant increase in glycolytic and TCA cycle genes.^50^ Also, a change in metabolism toward oxidative phosphorylation was suggested and the contribution of MS astrocytes to brain inflammation was hypothesized due to TCA cycle upregulation.^50^ In the study of in vitro oligodendrogliopathy model using human CNS-derived oligodendrocytes, the majority of ATP was generated in aerobic glycolysis that helps protein and lipid synthesis needed to form myelin.^51^ Reduced glycolytic ATP production would worsen myelin withdrawal and promote cell survival.^51^

### Reporter Metabolite Analysis: MS Study

Oligodendrocytes are the primary targets in MS, where the immune system attacks and damages the myelin they produce, leading to demyelination and disruption of nerve signal transmission. Astrocytes contribute to the formation of glial scars in areas of demyelination, which can inhibit repair processes and axonal regeneration. Microglia become activated in MS, contributing to inflammation and further damage to myelin and neurons through the release of proinflammatory cytokines and reactive oxygen species. Neurons can suffer secondary damage due to demyelination and inflammation, leading to axonal loss and neurodegeneration, which correlate with the progression of disability in MS. Thus, comprehending the functions and alterations of distinct brain cell types (oligodendrocytes, astrocytes, microglia, and neurons) is imperative in the advancement of tailored treatments intended to regulate the immune system, protect myelin, and stimulate repair in diseases such as MS.

In the present study, taking MS as a neurodegenerative disorder example, reporter metabolite analysis (RMA) was performed to understand metabolic reprogramming/regulation in response to disease-related changes in the brain. RMA identifies the key metabolites that are most significantly affected by transcriptional changes in their associated gene expressions in response to different perturbations.^52^ Our analysis revealed hot spots (important reporter metabolites) in microglia and oligodendrocytes (Supplementary Data 2). Regardless of their subcellular localization, 219 and 257 metabolites (p-value <0.05) were identified as reporter metabolites in GM and WM microglia, respectively. L-carnitine was found to be the top reporter metabolite in GM and arachidonate in the WM region. L-carnitine was only predicted in GM; however, arachidonate was predicted in both regions. Similar to L-carnitine, leukotriene E4 and N-acetyl-LTE4 were only identified as reporter metabolites in GM microglia. GM and WM samples have 82 common reporter metabolites and a large number of them including arachidonate, adrenic, behenic, decanoic, linoleic, palmitic, oleic, hexanoic, octanoic, and valeric acids were involved in fatty acid-related metabolites in both GM and WM microglia.

**Figure 8:**
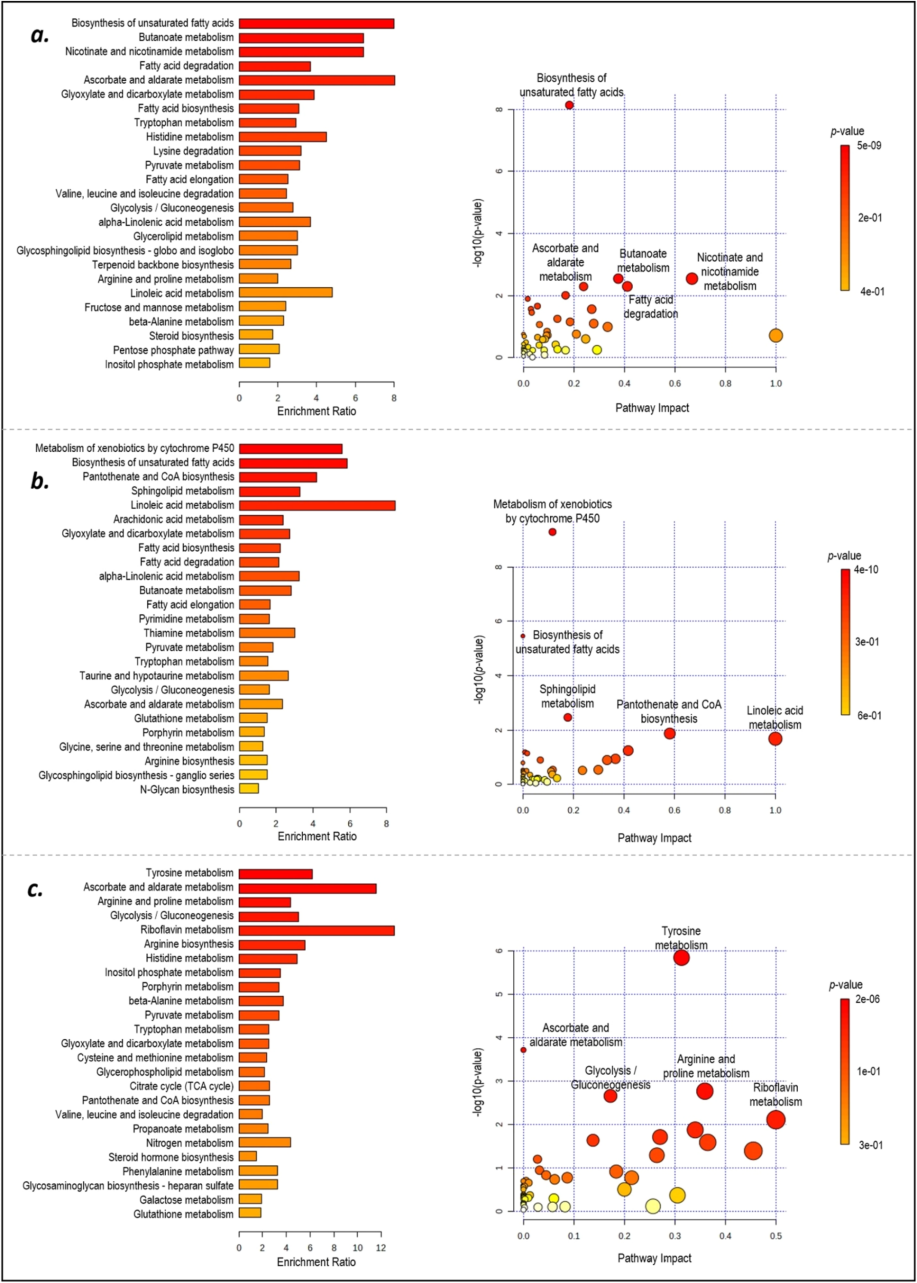
Over-representation and pathway analysis of reporter metabolites in (a) GM and (b) WM microglia and (c) oligodendrocytes due to MS.

Over-representation analysis (ORA) of reporter metabolites demonstrated that biosynthesis of unsaturated fatty acids is the most significantly over-represented one in GM microglia (Figure 8a) and metabolism of xenobiotics by cytochrome P450 in WM microglia (Figure 8b) in MS patients. Butanoate metabolism, nicotinate and nicotinamide metabolism, fatty acid degradation, ascorbate and aldarate metabolism, glyoxylate and dicarboxylate metabolism and fatty acid biosynthesis in GM microglia and biosynthesis of unsaturated fatty acids, pantothenate and CoA biosynthesis, sphingolipid metabolism, and linoleic acid metabolism in WM microglia were also significantly over-represented (*p*-value <0.025) in MS patients. ORA of common metabolites in gray and white matter MS pointed out significant enrichment (*p*-value <0.025) in the biosynthesis of unsaturated fatty acids, fatty acid biosynthesis, α-linolenic acid metabolism, butanoate metabolism, and fatty acid degradation (Figure S2).

In CNS, triggering receptor expressed on myeloid cells 2 (TREM2) regulating the cellular activity in microglia in the degradation of lipid-based components is highly expressed in microglia and is strongly found in demyelinating plaques in MS patients.^53^ A variety of lipid- and lipid-interacting ligands, such as sphingomyelin, oxidized phospholipids, phosphatidylcholine, and APOE, activate TREM2. The formation of sphingosine 1-phosphate (S1P), a ligand for the S1P receptor family produced by microglia, is stimulated by inflammatory lesions. The elimination of highly inflammatory and toxic small molecules that build up at demyelinating sites is made possible by receptor-mediated endocytosis. Meanwhile, microglial phagocytosis clears large molecular debris such as myelin and dead cells produced during lesion growth, which may otherwise hinder repair.^53^

Primary progressive multiple sclerosis (PPMS), a type of MS, leads to a continuous decline in brain and nerve function without significant periods of remission or relapse. RMA resulted in 122 reporter metabolites (*p*-value < 0.05) in oligodendrocytes (Supplementary Data 2). Carbamoyl-phosphate, acetate, and ornithine were computed as top reporter metabolites (*p*-value < 0.025). ORA of reporter metabolites demonstrated significant enrichment (*p*-value < 0.025) in tyrosine metabolism, ascorbate and aldarate metabolism, arginine and proline metabolism, glycolysis/gluconeogenesis, riboflavin metabolism, arginine biosynthesis, and histidine metabolism in PPMS oligodendrocyte (Figure 8c). Oligodendrocyte results have 26 and 11 common reporter metabolites with GM and WM microglia, respectively (Figure S2). Nine of them, including indoleacetate, d-glucarate, indole-3-acetaldehyde, 3-amino-propanal, and acetyl-CoA, are shared reporter metabolites in both oligodendrocyte and microglia MS.

Arachidonate was identified as a reporter metabolite in our study. The arachidonic acid is metabolized to protanoids, prostacyclin, and thromboxane via cyclooxygenases (COXs). Lipoxygenases (LOXs) convert arachidonic acids into leukotrienes and hydroxyeicosatetraenoic acids (HETEs). COX-2 and 5-LOX inhibitors provide a potential strategy in MS treatment by targeting the arachidonic acid pathway.^54^ Several short-, medium-, and long-chain fatty acids were found as reporter metabolites in MS. Fatty acids are in relation to critical metabolic processes such as immune cell polarization, differentiation, and function, blood–brain integrity, inflammation, degeneration, and remyelination.^55^ Supplementation with different fatty acids demonstrated alleviation of symptoms in MS animal models. Another reporter metabolite, identified in the present study, is L-carnitine, which is necessary for the transport of long-chain fatty acids into mitochondria. In a pilot study, serum carnitine level was detected significantly lower in MS patients with fatigue compared with patients without fatigue, and it was suggested that higher dietary carnitine intake may provide benefits for MS-related fatigue.^56^ In the cuprizone-induced demyelinating rat model study, L-carnitine treatment demonstrated the neuroprotective effect and significantly improved abnormalities in rat sciatic nerves.^57^

## Conclusions

The computational analysis gives clues about the metabolic behaviors in the human brain, which is one of the most complex organs in the body because of the interactive cellular metabolism of its specific cells including neurons and glial cells. Here, the reconstructed brain cell-specific (iNeuron2527, iAstrocyte2303, iMicroglia1540, iOligodendrocyte2023, and iOPC1795) and integrated brain (iHumanBrain2690) GEMs allowed us to investigate human brain metabolism at the system level. The metabolic fluxes in many subsystems of the core carbon, amino acid, and fatty acid metabolisms were predicted by FBA at the resting state. The changes in glucose, ketone bodies, and oxygen were observed to result in notable metabolic alterations in glycolysis/gluconeogenesis, the TCA cycle, and glyoxylate/dicarboxylate and oxidative phosphorylation subsystems and intercellular reactions.

Neurological diseases are considered critical perturbations leading to dysfunction of brain metabolism and cerebral activity. One of them is MS, a chronic disease of CNS, characterized by inflammation, demyelination, and axonal damage. Inflammatory microglia induce astrocytes to become highly reactive and neurotoxic. These reactive astrocytes can harm CNS by producing reactive oxygen species and saturated lipids and may disrupt metabolic regulation and glutamatergic signaling at synapses, resulting in excitotoxicity. Thus, the multifaceted nature of these neurological diseases requires the reconstruction of several single brain cell-specific GEMs, which further provide opportunities for researchers to reconstruct a two- or three-compartment integrated human brain network with the specific brain cells of interest to investigate metabolic interactions in and between them. Our simulations with the integrated metabolic brain GEM, iHumanBrain2690, are in agreement with the experimental results published in the literature, validating its correct reconstruction and allowing its use in the interpretation of key cell-specific characteristics of various metabolic states and diseases, such as intracranial tumors and neurodegenerative and neurodevelopmental disorders.

Our brain networks can also be combined with pathogen metabolic networks to decipher pathogen–host interactions in the human brain computationally. Consequently, single and integrated human brain GEMs can help in the elucidation of metabolic activities in health and neurological diseases for the development of novel therapeutic strategies. Experimental and clinical studies are needed for computational approaches, and collaborative studies may help expedite the development of effective treatment strategies.

## Materials and Methods

### Reconstruction of Brain Cell-Specific GEMs

GSE67835 which investigates transcriptional differences in human brain cells including neurons, astrocytes, microglia, oligodendrocytes, and oligodendrocyte precursor cells (OPCs) was obtained from the Gene Expression Omnibus database.^7,58^ GSE67835 covers single-cell RNA-seq of 466 cell samples from healthy brains. The data set is given with raw read values for each sample. Raw read values were normalized to transcript per million reads (TPM) using transcript lengths in the human genome from Lopes et al. where the gene lengths were calculated from the BioMart Web site based on the human reference genome (GRCh38.p12).^59,60^ The normalization is performed by taking both gene length and sequencing depth into consideration in the TPM calculation.^61^

Human1 metabolic network was used as a generic human GEM.^30^ The task-driven integrative network inference for tissues (tINIT) algorithm was selected to integrate Human1 with gene expression data to generate brain cell-specific GEMs.^31^ Mean values of TPM were calculated, and a cutoff value of 1 was used for the tINIT algorithm (Figure S3). The major advantage of the tINIT algorithm is that all predefined metabolic tasks are satisfied by the automatically generated GEMs. Robinson et al. (2020) defined 57 essential metabolic tasks that are necessary for human cell viability.^30^ In addition to predefined essential metabolic tasks, cell-specific metabolic tasks obtained from the literature for astrocytes, neurons, microglia, and oligodendrocytes were included in the GEM extraction via the tINIT algorithm.

The previous human brain models^19,62^ are valuable sources of metabolic tasks for astrocytes and neurons since they were manually reconstructed to investigate metabolic interactions in and between astrocytes and neurons using literature support. The production of glutamate from glutamine and the conversion of glutamate into 4-aminobutyrate (GABA) were added as metabolic tasks in neurons. The production of glutamine from glutamate was added to the astrocyte, and the conversion of GABA into glutamate was defined for both neuron and astrocyte compartments. The reaction of serine into glycine and its reverse reaction were added to neurons and astrocytes, respectively. The conversion of branched-chain amino acids (leucine, isoleucine, and valine) was defined in both neuron and astrocyte lists to form α-ketoisocaproate, α-keto-β-methylvalerate, and α-keto-isovalerate, respectively. Dopamine production from levodopa was defined in neurons, and its conversion into norepinephrine was included in both astrocyte and neuron metabolic tasks. Adrenaline and acetylcholine productions were added to the neuron list.

The conversion of glutamine into glutamate was included in microglia GEM reconstruction.^25^ N-acetyl-l-aspartate (NAA) synthesis was defined in neurons and its degradation was added to the oligodendrocyte metabolic list.^63^ The main idea behind defining additional brain cell-specific metabolic tasks is to include related reactions and their metabolites (neurotransmitters) in the integration and crosstalk of brain-specific cells. Different from other tasks, myelin sheath formation reaction, considered as essential metabolic task in oligodendrocytes, does not exist in Human1, which is used as a generic human model with the tINIT algorithm. Therefore, the myelin formation reaction was defined by its major components including cholesterol, galactosylceramide, phosphatidylcholine, phosphatidylethanolamine, and sphingomyelin.^64^ Along with its exchange and transport reactions, it was added to the Human1 model before the oligodendrocyte-specific GEM reconstruction. Additional metabolic tasks were not defined for OPCs and its GEM was generated with 57 essential metabolic tasks.

### Integration of Neuron, Astrocyte, Microglia, and Oligodendrocyte GEMs into iHumanBrain2690

To understand the metabolic interaction in the human brain among neurons, astrocytes, microglia, and oligodendrocytes, four brain cell type-specific GEMs (iNeuron2527, iAstrocyte2303, iMicroglia1540, and iOligodendrocyte2023) were integrated with the intercellular exchange reactions. To label reactions, subsystems, subcellular compartments, and metabolites, “_*N*,″ “_A,” “_M,” and “_O” suffixes were used for neuron, astrocyte, microglia, and oligodendrocyte compartments, respectively. Subsequent to the conversion of RAVEN-compatible GEMs into COBRA-compatible structures via “ravenCobraWrapper” function, the integration was performed by the “mergeTwoModels” function in the COBRA Toolbox.^65,66^ First, neuron and astrocyte models were combined, and then, microglia and oligodendrocyte models were sequentially added to this two-compartment model to generate a four-compartment brain model. The reactions representing intercellular neurotransmitter exchange and metabolic crosstalk (Table S5) among these brain cells were added based on available human brain GEMs and literature support using “addReaction” function. In addition to intercellular reaction available in iMS570,^19^ glutamine transfer from astrocyte to microglia and glutamate transfer from neuron to microglia were defined.^67^ Intercellular lactate transport among these four brain cells and NAA transfer from neuron to oligodendrocyte were added in accordance with the literature.^25,68^

### Flux Balance Analysis (FBA), Flux Variability Analysis (FVA), and Minimization of Metabolic Adjustment (MOMA)

FBA and FVA were used to predict metabolic fluxes in single-cell (iNeuron2527, iAstrocyte2303, iMicroglia1540, iOligodendrocyte2023, and iOPC1795) and integrated (iHumanBrain2690) metabolic networks in healthy-state human brain using IBM Cplex Optimizer in MATLAB.^69,70^ The constraints (Table S6) were obtained from previous human brain metabolic networks for neurons and astrocytes and implemented by setting upper and lower flux limits.^19,62^ Pentose phosphate pathway fluxes were set to 5% and 6% of the glucose uptake flux for neurons and astrocytes, respectively. Microglia and oligodendrocytes, similar to astrocytes, are also classified as glial cells. Therefore, the upper bound flux constraints for microglia and oligodendrocytes were used the same as astrocyte constraints and their lower flux bounds were set to zero (Table S6). To avoid free metabolite uptake and infeasible solution, the lower bound of the rest of the exchange reactions was set to −0.01 μmol/g tissue/min and the upper bounds were set to 1000 μmol/g tissue/min not to limit metabolite release out of the cell. Maximization of biomass reaction fluxes was used as the first objective function in all single brain cell simulations in FBA. Owing to the possibility of multiple optima, minimization of the squared sum of intracellular fluxes was carried out as the second optimization by fixing the maximum value of biomass reaction flux obtained from the first optimization. Mean subsystem fluxes, number of reactions carrying nonzero fluxes (flux value >10^–6^ μmol/g tissue/min), and total number of reactions in the related subsystem were visualized using the SRplot online tool.^71^ FVA was performed by minimizing and maximizing the flux through each reaction to determine the range of possible reaction fluxes.^70^

In the iHumanBrain2690 metabolic network, maximization of the glutamate/glutamine/GABA cycle between neuron and astrocyte was used as the objective function in FBA.^19,62^ The objective value was added to the stoichiometric matrix, and the second quadratic optimization was carried out to obtain objective values with minimum fluxes. Intercellular GABA flux between the neuron and astrocyte was set to 25% of glutamate–glutamine cycle flux. The exchange reaction fluxes estimated from single-cell simulations were used as constraints in the flux analysis of an integrated human brain model. Oligodendrocytes with active myelin formation (flux value of 0.01 μmol/g tissue/min) were used in the integrated brain model analysis. To simulate metabolic changes due to demyelination, myelin reaction flux was reduced from 0.010 μmol/g tissue/min to zero, and the MOMA approach was applied by minimizing the Euclidean distance between the flux distributions.^72^ For the *in silico* analysis of metabolic alterations in the ketogenic diet via iHumanBrain2690, acetoacetate and (R)-3-hydroxybutanoate uptake fluxes of four brain cells were increased to 0.050 μmol/g tissue/min, and glucose was increased to 0.210 μmol/g tissue/min. For cerebral hypoxia, oxygen uptake fluxes were decreased by 0.100 μmol/g tissue/min. Subsystem activities in the four single brain cells in iHumanBrain2690 were calculated as the sum of the absolute value of metabolic fluxes in the corresponding subsystem. Glucose and ketone body supplemented and oxygen reduction conditions were compared with the resting state.

### Reporter Metabolite Analysis (RMA)

Two different gene expression data sets (GSE111972 and GSE211739) were obtained from the Gene Expression Omnibus (GEO) database to elucidate reporter metabolites in microglia and oligodendrocytes due to MS.^52,58^ GSE111972 covers the transcriptional profile of human microglia in gray matter (GM) and white matter (WM) regions in MS.^9^ GSE211739 includes transcriptional changes of mature oligodendrocytes which were differentiated from human-induced pluripotent stem cell lines derived from primary progressive MS and healthy donors.^73^ The MS microglia and oligodendrocyte samples were statistically compared with the corresponding control samples. The p-values were computed for each gene. The results were used with iMicroglia1540 and iOligodendrocyte2023 GEMs for the identification of reporter metabolites in microglia and oligodendrocytes due to MS. The computation was performed by the “reporterMetabolites” function in the RAVEN toolbox.^65^ The reporter metabolites (*p*-value < 0.05) were used in metabolite set enrichment and pathway topology analyses based on KEGG human metabolic pathways via MetaboAnalyst 6.0, which is one of the most commonly used tools for metabolomics data analysis.^74,75^ Hypergeometric test was applied in the enrichment analysis and relative-betweenness centrality in pathway topology analysis.

## Supporting Information Available

Table S1: Metabolic task performances of generated GEMs; Table S2: Maximum values of biomass reaction fluxes at resting state; Table S3: Mean subsystems fluxes and number of reactions in transport and exchange/demand reactions subsystems; Table S4: Number of reactions with nonzero flux in iHumanBrain2690 simulation; Table S5: Intercellular reactions among astrocyte, neuron, microglia and oligodendrocyte in iHumanBrain2690; Table S6: Cerebral metabolic rates used as constraints for the simulation of resting state flux distributions; Figure S1: Change of oligodendrocyte biomass reaction flux with myelin formation reaction flux; Figure S2: (a) Common reporter metabolites in MS microglia and oligodendrocytes (b) ORA of shared reporter metabolites in grey and white matter microglia; Figure S3: The distribution of TPM normalized gene expression used in the GEM generation via tINIT algorithm for astrocyte, neuron, microglia, oligodendrocyte, and OPC (PDF)
Supplementary Data 1: Human brain GEMs and flux distributions (ZIP)
Supplementary Data 2: Reporter metabolites (ZIP)

## Abbreviations

AD: Alzheimer’s disease
CNS: central nervous system
COX: cyclooxygenase
FBA: flux balance analysis
FVA: flux variability analysis
GABA: 4-aminobutyrate
GEM: genome-scale metabolic model
GEO: gene expression omnibus
GM: gray matter
HETE: hydroxyeicosatetraenoic acid
HMG-CoA: 3-hydroxy-3-methylglutaryl-CoA
LOX: lipoxygenase
LTE4: leukotriene E4
MOMA: minimization of metabolic adjustment
MS: multiple sclerosis
NAA: N-acetyl-l-aspartate
OPC: oligodendrocyte precursor cell
ORA: over-representation analysis
PPMS: primary progressive multiple sclerosis
RMA: reporter metabolite analysis
S1P: sphingosine 1-phosphate
TCA: tricarboxylic acid
tINIT: task-driven integrative network inference for tissues
TPM: transcript per million reads
TREM2: triggering receptor expressed on myeloid cells 2
WM: white matter
