# Supplementary material for: Exploring Human Brain Metabolism via Genome-Scale Metabolic Modeling with Highlights on Multiple Sclerosis
Source: ACS Chem Neurosci. 2025 Mar 17;16(7):1346–60. doi: 10.1021/acschemneuro.5c00006 (PMC11969529; doi:10.1021/acschemneuro.5c00006)
Supplement: Supplementary file 1 — cn5c00006_si_001.pdf [file cn5c00006_si_001.pdf]

# Exploring human brain metabolism via genome-scale metabolic modeling with highlights on multiple sclerosis

Mustafa Sertbas[1,2] and Kutlu O. Ulgen[1*]

[1]Department of Chemical Engineering, Bogazici University, 34342 Istanbul, Turkey
[2]Department of Chemical Engineering, Istanbul Technical University, 34469 Istanbul, Turkey

* Corresponding author
Email: ulgenk@bogazici.edu.tr

Table S1. Metabolic task performances of generated GEMs

| **GEM** | **Total number of performed task** |
|---|---|
| iNeuron2527 | 218 |
| iAstrocyte2303 | 214 |
| iMicroglia1540 | 169 |
| iOligodendrocyte2023 | 202 |
| iOPC1795 | 179 |

Table S2. Maximum values of biomass reaction fluxes at resting state (µmol/g tissue/min)

| ***GEMs*** | Maximum values of biomass reaction fluxes (µmol/g tissue/min) | Number of reactions with nonzero flux (flux value > $10^{-6}$ µmol/g tissue/min) |
|---|---|---|
| ***iNeuron2527*** | 0.0178 | 1664 |
| **iAstrocyte2303** | 0.0114 | 1354 |
| ***iMicroglia1540*** | 0.0064 | 944 |
| ***iOligodendrocyte2023*** (inactive myelin formation) (myelin= 0) | 0.0422 | 1061 |
| ***iOligodendrocyte2023*** (active myelin formation) myelin= 0.0100 | 0.0279 | 1157 |
| ***iOPC1795*** | 0.0433 | 1216 |

Table S3. Mean subsystems fluxes and number of reactions in transport and exchange/demand reactions subsystems

| ***GEMs*** | Subsystems | Mean subsystems fluxes (μmol/g tissue/min) | Number of reactions with nonzero flux (flux value > 10-6 μmol/g tissue/min) | Total number of reactions |
|---|---|---|---|---|
| ***iNeuron2527*** | Transport reactions | 0.0152 | 614 | 2349 |
| | Exchange/demand reactions | 0.0270 | 257 | 913 |
| **iAstrocyte2303** | Transport reactions | 0.0152 | 452 | 2021 |
| | Exchange/demand reactions | 0.0166 | 198 | 805 |
| ***iMicroglia1540*** | Transport reactions | 0.0097 | 328 | 1444 |
| | Exchange/demand reactions | 0.0131 | 133 | 744 |
| ***iOligodendrocyte2023*** (inactive myelin formation) (myelin flux= 0) | Transport reactions | 0.0502 | 341 | 1897 |
| | Exchange/demand reactions | 0.0140 | 199 | 794 |
| ***iOligodendrocyte2023*** (active myelin formation) (myelin flux= 0.0100 μmol/g tissue/min) | Transport reactions | 0.0459 | 377 | 1897 |
| | Exchange/demand reactions | 0.0164 | 220 | 794 |
| ***iOPC1795*** | Transport reactions | 0.0406 | 312 | 1684 |
| | Exchange/demand reactions | 0.0275 | 182 | 761 |

Table S4. Number of reactions with nonzero flux in iHumanBrain2690 simulation

| ***Compartment in iHumanBrain2690*** | **Number of total reactions** | **Number of reactions with nonzero flux* at 0.010 µmol/g tissue/min myelin formation flux** |
|---|---|---|
| *Neuron* | 8004 | 1674 |
| *Astrocyte* | 7401 | 1336 |
| *Microglia* | 5565 | 916 |
| *Oligodendrocyte* | 6619 | 1195 |
| *Intercellular* | 26 | 10 |
| *iHumanBrain2690* | 27615 | 5131 |

*(flux value > $10^{-6}$ µmol/g tissue/min)

Table S5. Intercellular reactions among astrocyte, neuron, microglia and oligodendrocyte in iHumanBrain2690

| ***Reaction Name*** | ***Reaction Formula*** |
|---|---|
| ***Glutamine_A_to_N*** | glutamine_A [c_A] -> glutamine_N [c_N] |
| ***Glutamate_N_to_A*** | glutamate_N [c_N] -> glutamate_A [c_A] |
| ***GABA_N_A*** | 4-aminobutyrate_N [c_N] <=> 4-aminobutyrate_A [c_A] |
| ***Serine_A_to_N*** | serine_A [c_A] -> serine_N [c_N] |
| ***Glycine_N_to_A*** | glycine_N [c_N] -> glycine_A [c_A] |
| ***Leucine_N_to_A*** | leucine_N [c_N] -> leucine_A [c_A] |
| ***Isoleucine_N_to_A*** | isoleucine_N [c_N] -> isoleucine_A [c_A] |
| ***Valine_N_to_A*** | valine_N [c_N] -> valine_A [c_A] |
| ***KIC_A_to_N*** | 4-methyl-2-oxopentanoate_A [c_A] -> 4-methyl-2-oxopentanoate_N [c_N] |
| ***KMV_A_to_N*** | 2-oxo-3-methylvalerate_A [c_A] -> 2-oxo-3-methylvalerate_N [c_N] |
| ***KIV_A_to_N*** | 3-methyl-2-oxobutyrate_A [c_A] -> 3-methyl-2-oxobutyrate_N [c_N] |
| ***Dopamine_N_to_A*** | dopamine_N [c_N] -> dopamine_A [c_A] |
| ***Aspartate_N_A*** | aspartate_N [c_N] <=> aspartate_A [c_A] |
| ***Alanine_N_A*** | alanine_N [c_N] <=> alanine_A [c_A] |
| ***Cholesterol_A_to_N*** | cholesterol_A [c_A] -> cholesterol_N [c_N] |
| ***Arachidonate_A_to_N*** | arachidonate_A [c_A] -> arachidonate_N [c_N] |
| ***Decosahexenoate_A_to_N*** | DHA_A [c_A] -> DHA_N [c_N] |
| ***CysteinylGlycine_A_to_N*** | cys-gly_A [c_A] -> cys-gly_N [c_N] |
| ***Putrescine_N_to_A*** | putrescine_N [c_N] -> putrescine_A [c_A] |
| ***Glutamine_A_to_M*** | glutamine_A [c_A] -> glutamine_M [c_M] |
| ***Glutamate_N_to_M*** | glutamate_N [c_N] -> glutamate_M [c_M] |
| ***Lactate_A_to_N*** | L-lactate_A [c_A] -> L-lactate_N [c_N] |
| ***Lactate_A_to_O*** | L-lactate_A [c_A] -> L-lactate_O [c_O] |
| ***Lactate_O_to_N*** | L-lactate_O [c_O] -> L-lactate_N [c_N] |
| ***Lactate_M_to_N*** | L-lactate_M [c_M] -> L-lactate_N [c_N] |
| ***NAA_N_to_O*** | N-acetyl-L-aspartate_N [c_N] -> N-acetyl-L-aspartate_O [c_O] |

Table S6. Cerebral metabolic rates used as constraints for the simulation of resting state flux distributions (μmol/g tissue/min)

| ***GEMs*** | ***iNeuron2527*** | ***iAstrocyte2303*** | ***iMicroglia1540 iOligodendrocyte2023 iOPC1795*** |
|---|---|---|---|
| ***Constraints*** (μmol/g tissue/min) | Glucose: 0.16<br>$O_2$: 1.23<br>$CO_2$*: 1.193-1.230<br>Phenylalanine: 0.0132<br>Tryptophan: 0.0082<br>Tyrosine: 0.0041<br>Lysine: 0.0103<br>Histidine: 0.0025<br>Ornithine: 0.0031<br>Methionine: 0.0017<br>Threonine: 0.0008 | Glucose: 0.16<br>$O_2$: 0.53<br>$CO_2$*: 0.515-0.530<br>Cystine: 0.0045<br>Ammonia: 0.0035<br>Leucine: 0.0145<br>Isoleucine: 0.004<br>Valine: 0.0018<br>Arginine: 0.0020<br>Ornithine: 0.0031<br>Methionine: 0.0017<br>Threonine: 0.0008 | Glucose: 0.16<br>$O_2$: 0.53<br>Cystine: 0-0.0045<br>Ammonia: 0-0.0035<br>Leucine: 0-0.0145<br>Isoleucine: 0-0.004<br>Valine: 0-0.0018<br>Arginine: 0-0.0020<br>Ornithine: 0-0.0031<br>Methionine: 0-0.0017<br>Threonine: 0-0.0008 |

* $CO_2$ is release flux, others are uptake upper and lower bounds

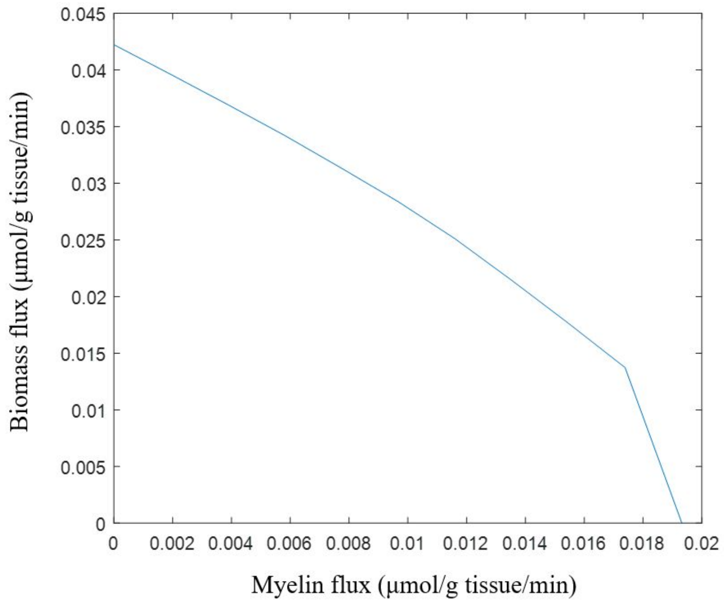


Figure S1. Change of oligodendrocyte biomass reaction flux with myelin formation reaction flux

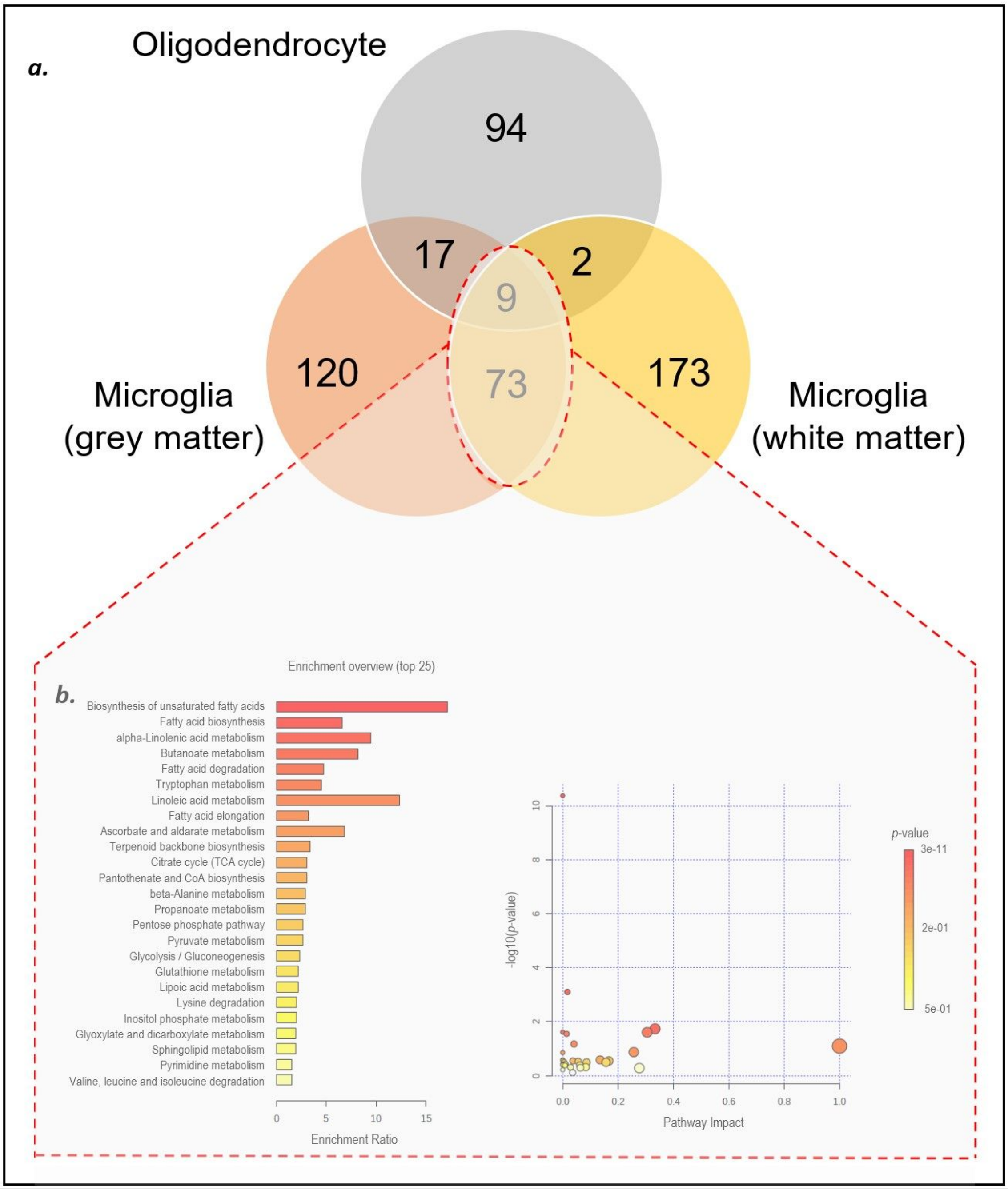


Figure S2. (a) Common reporter metabolites in MS microglia and oligodendrocytes (b) ORA of shared reporter metabolites in grey and white matter microglia

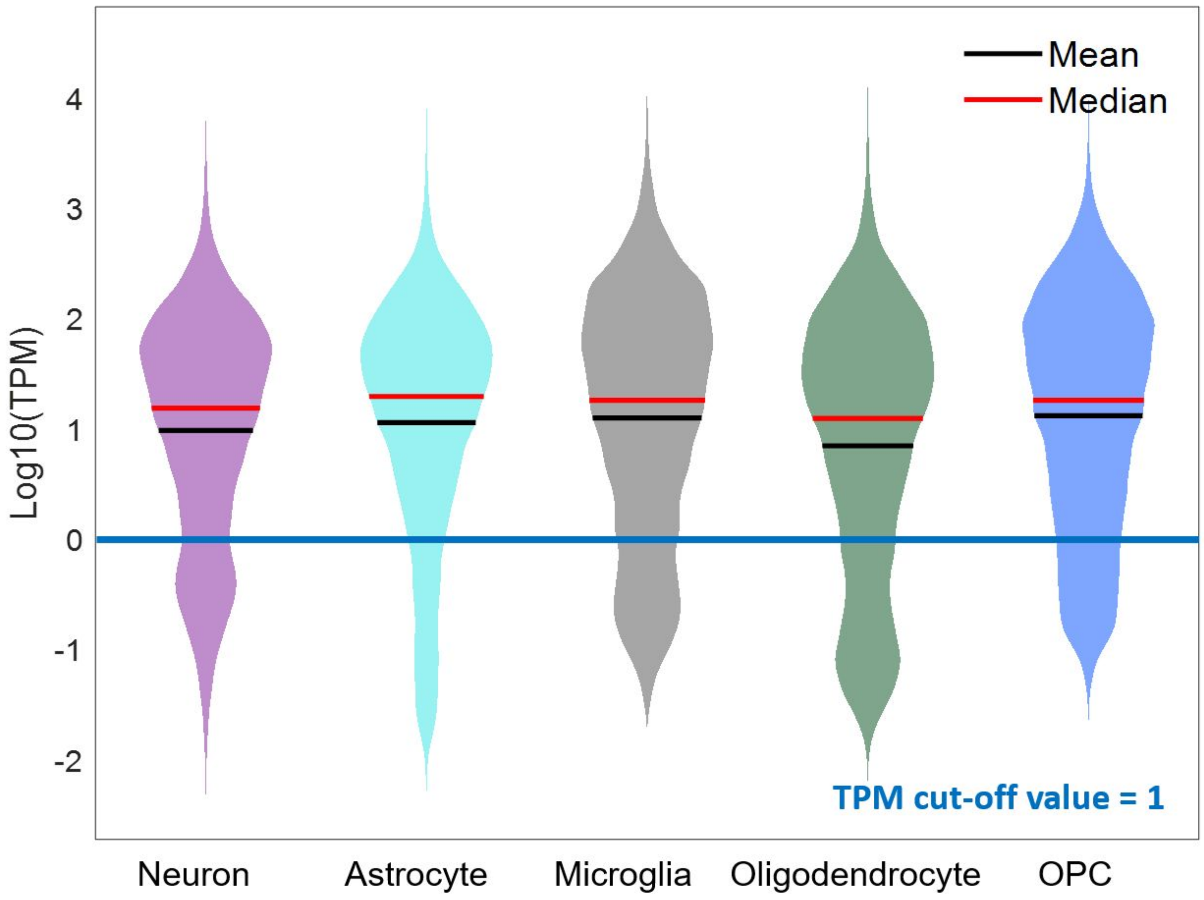


Figure S3. The distribution of TPM normalized gene expression used in the GEM generation via tINIT algorithm for astrocyte, neuron, microglia, oligodendrocyte, and OPC
